# Plasticity during Sleep Is Linked to Specific Regulation of Cortical Circuit Activity

**DOI:** 10.3389/fncir.2017.00065

**Published:** 2017-09-15

**Authors:** Niels Niethard, Andrea Burgalossi, Jan Born

**Affiliations:** ^1^Institute of Medical Psychology and Behavioral Neurobiology, University of Tübingen Tübingen, Germany; ^2^Center for Integrative Neuroscience, University of Tübingen Tübingen, Germany

**Keywords:** sleep, plasticity, excitation, inhibition, REM, SWS

## Abstract

Sleep is thought to be involved in the regulation of synaptic plasticity in two ways: by enhancing local plastic processes underlying the consolidation of specific memories and by supporting global synaptic homeostasis. Here, we briefly summarize recent structural and functional studies examining sleep-associated changes in synaptic morphology and neural excitability. These studies point to a global down-scaling of synaptic strength across sleep while a subset of synapses increases in strength. Similarly, neuronal excitability on average decreases across sleep, whereas subsets of neurons increase firing rates across sleep. Whether synapse formation and excitability is down or upregulated across sleep appears to partly depend on the cell’s activity level during wakefulness. Processes of memory-specific upregulation of synapse formation and excitability are observed during slow wave sleep (SWS), whereas global downregulation resulting in elimination of synapses and decreased neural firing is linked to rapid eye movement sleep (REM sleep). Studies of the excitation/inhibition balance in cortical circuits suggest that both processes are connected to a specific inhibitory regulation of cortical principal neurons, characterized by an enhanced perisomatic inhibition via parvalbumin positive (PV+) cells, together with a release from dendritic inhibition by somatostatin positive (SOM+) cells. Such shift towards increased perisomatic inhibition of principal cells appears to be a general motif which underlies the plastic synaptic changes observed during sleep, regardless of whether towards up or downregulation.

## Introduction

Research of the last decades has identified sleep as a key contributor to the continuing changes the brain undergoes while adapting to its environment. Basically, two different functions of sleep have been revealed in this context: on the one hand, sleep was shown to serve the consolidation of memories and, thus, to support synaptic plasticity in memory-specific circuitry mediating these consolidation processes (Diekelmann and Born, 2010; Rasch and Born, 2013). On the other hand, sleep was shown to support the global homeostatic regulation of synaptic turnover in the brain (Tononi and Cirelli, 2014). In the sleeping brain both of these functions might be established in a co-operative manner, partly relying on the same regulatory mechanisms. Encoding and learning in the waking brain implies that neuronal circuits continuously adapt their connections in respect to the incoming inputs. Connections are selectively strengthened or weakened, while the overall numbers of potentiated synapses are increasing across the wake period. During sleep the temporally limited disconnection from the outer world provides unique opportunities to rearrange and rescale existing neural connections and representations, both locally for specific memory representations and globally. Indeed, computational approaches indicate that synaptic rescaling during the offline period of sleep is not only a prerequisite for maintaining synaptic homeostasis, but can also produce substantial improvements of neuronal networks in overcoming catastrophic forgetting during sequential learning processes (Olcese et al., 2010; Hashmi et al., 2013; Nere et al., 2013; Kirkpatrick et al., 2017). Here, we briefly summarize recent evidence indicating that sleep rescales synaptic strength within neural networks thereby presumably supporting both processes, i.e., the consolidation of specific memories as well as the maintenance of global synaptic homeostasis. Moreover, we propose that synaptic rescaling during sleep is generally linked to a unique regulation of the excitation/inhibition balance within cortical circuits. A main feature of this regulation is a shift in the inhibitory control of excitatory cells from dendritic inhibition—mediated by somatostatin positive (SOM+) interneurons—towards predominant perisomatic inhibition—mediated by parvalbumin positive (PV+) interneurons. In the following, we first briefly summarize evidence on how sleep affects structural measures of synaptic turnover and neuronal excitability and excitation, and then focus on the dynamics of excitation/inhibition balance within cortical circuits during sleep. We will also point out sleep stage specific effects as far as the studies provide respective information.

## Sleep and Structural Synaptic Changes

Whereas encoding of information in the waking brain manifests itself mainly in the potentiation of selected synapses, the formation of persisting memories and the long-term maintenance of synaptic upregulation are often associated with characteristic structural and morphological changes at the involved synapses. Such changes can occur well within several hours after encoding of the information, and might be related to the transition from short-term to long-term memory (Hofer et al., 2009; Xu et al., 2009; Yang et al., 2009). Correspondingly, processes in the opposite direction like the shrinking and pruning of synaptic contacts might be linked to some kind of forgetting but, alternatively, might merely reflect the homeostatic regulation of synaptic turnover such that upscaling of some synapses requires downscaling of others.

The first imaging approaches using *in vivo* two-photon microscopy revealed a net loss of synapses during sleep in the developing mouse cortex (Maret et al., 2011; Yang and Gan, 2012) and in the mushroom bodies of fruit flies (Bushey et al., 2011). A recent study using three dimensional electron microscopy showed that during sleep the area of contact between axon terminals and dendritic spines of cortical synapses was decreased on average by 18% (de Vivo et al., 2017). Detailed analyses revealed that the decrease in axon-spine interface area was not equally distributed across different synapses, but spared the top 20% of the biggest (and possibly strongest) synapses. Evidence for downscaling effects of sleep were also revealed in a study investigating spine size and AMPA receptor densities in the mouse cortex (Diering et al., 2017) with the latter measure known to strongly correlate with synaptic strength (Matsuzaki et al., 2001; Béïque et al., 2006; Zito et al., 2009). Sleep induced a global reduction in spine size and a weakening of synapses through the removal and dephosphorylation of AMPA-type glutamate receptors. However, in contrast to the previous study (de Vivo et al., 2017) the spines with more AMPA receptors—presumably the strongest synapses—were more likely to lose AMPA receptors and shrink in size during sleep. Importantly, whereas sleep globally reduced levels of AMPA receptors (Vyazovskiy et al., 2008), a small fraction of spines showed increased AMPA receptor levels after sleep. This small fraction of spines likely represents information encoded during prior wakefulness. This view would be also in line with monocular deprivation experiments which showed upregulation of the phosphorylation levels within the visual cortex of the AMPA receptor subunit GluR1 during subsequent sleep (Aton et al., 2009). There is also evidence suggesting that sleep-dependent regulation of AMPA receptors differentially affects AMPA receptor subtypes, with the elimination and upregulation of receptor levels mainly pertaining to calcium permeable AMPA receptors which do not contain the GluR2 subunit and keep the circuit in a labile state (Lanté et al., 2011; Shepherd, 2012; Del Cid-Pellitero et al., 2017).

There is also initial evidence supporting a differential role of slow wave sleep (SWS) and rapid eye movement (REM) sleep in the regulation of synapse morphology. In mice, training of a motor task was followed by the reactivation of layer 5 pyramidal cells in motor cortex during SWS, which was accompanied by branch-specific spine formation on the apical dendrites of these cells (Yang et al., 2014). These effects, however, were not disrupted by selective deprivation of REM sleep during the sleep period following motor learning. A second study from the same group (Li et al., 2017) elucidated the contributions of REM sleep to the regulation of synapse turnover, applying also REM sleep deprivation after motor task learning in mice. REM sleep was found to promote the elimination of newly formed spines, and this REM sleep dependent elimination subsequently facilitated spine formation when a new task was learned after sleep. Interestingly, the task-related reactivation of branch-specific Ca^2+^ spikes during REM sleep stabilized the newly-formed synaptic connections by preventing their pruning. These observations are in line with findings in awake mice where task-related dendritic Ca^2+^ spikes were found to cause long-lasting spine potentiation during learning of a motor task (Cichon and Gan, 2015). Altogether, these studies point to a general role of dendritic Ca^2+^ spikes in mediating lasting synapse formation, which is independent of the brain state.

In sum, these initial studies consistently support a global down-scaling effect of sleep on structural measures of synapse formation and efficacy, whereby it is not yet clear whether the strongest synapses are more or less affected by this downscaling. Concurrently, a small proportion of synapses appears to be spared from downscaling or is even up-scaled during sleep. Monitoring of task-related synaptic networks provided the first indications that the memory-specific up-scaling of synapses in dendritic branches of layer 5 pyramidal neurons occurs during SWS, whereas the elimination of newly formed spines in branches not undergoing task-specific reactivation is supported by REM sleep.

## Sleep’s Effect on Neuronal Excitability

How the strength of synapses relates to neural firing rates is very well described by the concept of synaptic rescaling (Turrigiano et al., 1998; Turrigiano, 2012; Hengen et al., 2016). This concept, based mainly on *in vitro* experiments, assumes that the total strength of synaptic inputs on a neuron is regulated such that firing rates remain at a fairly constant level. Accordingly, when encoding of information produces a potentiation of selected synaptic inputs of a specific neuron which, consequently, leads to increased firing activity of this neuron, then this initiates a number of counter-regulatory processes that eventually return firing rates to the initial levels. If the information encoded is maintained, then the relative differences in strength of the neuron’s synapses are also maintained during this rescaling of synapses. The counter-regulatory processes can occur at a slow pace in the individual neuron with baseline firing rates earliest recovered after some hours, but they are also established at the level of circuits and networks, and at the circuit level baseline firing in the target neuron might be achieved at a much faster rate (Turrigiano, 2017).

Over the course of wakefulness, the excitability of cortical neurons has been shown to increase, in line with the idea that during waking, neuronal networks are constantly shaped by new experience that is incorporated into the existing network by establishing stronger connections between neurons (e.g., Vyazovskiy et al., 2008; Huber et al., 2013; Piantoni et al., 2013; Tononi and Cirelli, 2014; Miyawaki and Diba, 2016). Conversely over the course of sleep, cortical excitability globally decreases. This is indicated by neuronal firing rate measurements in rodents (Vyazovskiy et al., 2009; Grosmark et al., 2012; Miyawaki and Diba, 2016) and by measurements of cortical excitability through transcranial magnetic stimulation (TMS) in humans (Civardi et al., 2001; Kreuzer et al., 2011). A global decrease in excitability across sleep is also suggested by recordings of EEG and local field potential in humans and rodents indicating a decrease in synchrony of potential activity, as measure of network synaptic connectivity, across sleep. Accordingly, after prolonged periods of wakefulness, EEG slow-wave activity and the incidence of slow oscillations (SO) during subsequent SWS is enhanced, whereas these measures decrease over the course of sleep (Huber et al., 2000; Riedner et al., 2007; Vyazovskiy et al., 2011; Nelson et al., 2013; Korf et al., 2017).

Notably, the observed global decrease in excitability across sleep appears to be differentially regulated by SWS and REM sleep. Studies in rodents have shown that on average, neocortical and hippocampal neurons decrease firing activity on average across SWS-REM sleep-SWS triplets (Grosmark et al., 2012; Watson et al., 2016) suggesting a global decrease in activity induced during REM sleep. However, within the REM sleep epochs themselves, firing dynamics appeared to be mixed, with many cells increasing in activity. Analysis of triplets of SWS-REM sleep-SWS epochs revealed the decreases in hippocampal neural firing across REM sleep epochs to be positively correlated with the power of theta oscillations (4–12 Hz) during the intermittent REM sleep epoch (Grosmark et al., 2012). The notion that REM sleep globally downregulates excitability in widespread neural networks is also corroborated by a TMS study in humans showing increased cortical excitability after selective REM sleep deprivation (Placidi et al., 2013).

By contrast, the role of SWS in the regulation of excitability appears to be more complex. On average, hippocampal principal cells increased firing activity in the course of a single SWS episode (Grosmark et al., 2012). The dynamics of frontal cortical neurons during SWS depended on their activity level during wakefulness (Watson et al., 2016) i.e., the neurons most active during wakefulness showed a substantial fall in activity during SWS, whereas the neurons least active during wakefulness increased in activity. The latter observation is of special interest, because in hippocampal networks, the low firing cells are the ones that are more likely to be implicated in experience-dependent plasticity and memory encoding, i.e., to develop a precise place field coding when a rat is exploring a new environment. And these cells also show increased temporal co-activation (i.e., replay) during succeeding SWS (Grosmark and Buzsáki, 2016). However, mechanisms of plasticity distinctly differ between hippocampus and neocortex (e.g., Frankland et al., 2001; Lee and Kirkwood, 2011), and future studies need to proof whether this specific relationship also holds for cortical neurons.

**Figure 1:**
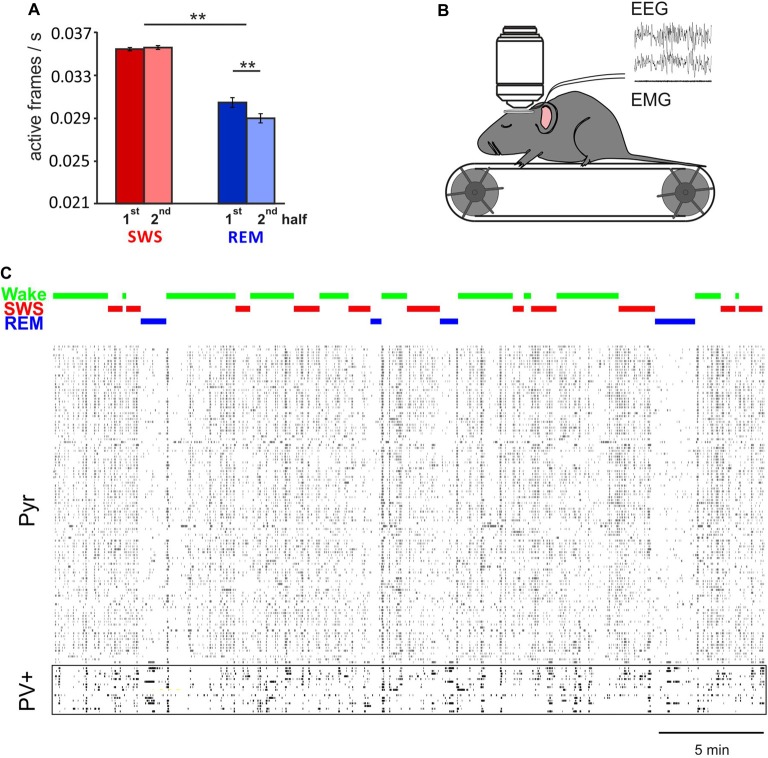
*In vivo* two-photon Ca^2+^ imaging during sleep.** (A)** Mean (±SEM) activity of pyramidal cells during the first and second half of all slow wave sleep (SWS) and rapid eye movement (REM) sleep episodes. Note, during REM sleep activity is overall lower than during SWS. Activity decreases within REM sleep epochs, but remains at the same level during SWS epochs. **p < 0.01 for pairwise comparisons. **(B)** *In vivo* two-photon imaging was performed on head-fixed mice (expressing GCaMP6f, while parvalbumin positive (PV+) or somatostatin positive (SOM+) cells are labeled with tdTomato, for recording of putative Pyr, PV+ and SOM+ cells respectively) which repeatedly went through periods of wake, SWS and REM sleep during one imaging session. Sleep stages were identified by EEG and EMG. **(C)** Example recordings of activity in 110 putative pyramidal cells and 17 PV+ cells that were imaged simultaneously. Note, activity of pyramidal cells, but not PV+ cells, is substantially decreased during REM sleep (with permission from Elsevier adapted from Niethard et al., 2016).

Imaging of Ca^2+^ activity in cortical pyramidal cells only partly confirmed the electrophysiological studies. Namely, unlike electrophysiological analyses of spike firing rates (e.g., Vyazovskiy et al., 2009) in cortical layer 2/3 we found generally lower, rather than higher activity of pyramidal cells during REM sleep, in comparison with SWS and wakefulness. The reasons for this discrepancy are not clear (see Niethard et al., 2016). It might reflect that electrophysiological recordings were performed in different layers (mostly layer V) but also, that the calcium signal is not an immediate reflection of discrete action potential activity (Chen et al., 2013; Badura et al., 2014). In line with previous electrophysiological findings (Grosmark et al., 2012), Ca^2+^ imaging indicated that pyramidal cell activity on average decreased within REM sleep epochs but not within SWS epochs (Figure 1). Clustering cells according to their average activity level during wake epochs, did not reveal a consistent pattern, as to whether the cell underwent down or upregulation of activity during SWS. Across the total sleep epoch, activity on average appeared to decrease.

Taken together, currently-available data consistently indicate that on average, sleep downregulates excitability of cortical and hippocampal networks. REM sleep appears to contribute to this effect in a robust and global manner, whereas SWS appears to contribute in a more differential manner. An intriguing finding is that cells which are less active during wakefulness, and which are more likely implicated in experience-dependent plasticity, increase their activity across SWS, and ultimately across the entire sleep epoch. These differential dynamics, moreover, appear to differ for hippocampal and neocortical neurons, which overall tempts to speculate that neural activity dynamics during SWS, to a greater extent than during REM sleep, reflect processes of memory consolidation.

## Excitation/Inhibition Balance During Sleep—Distinct Roles for PV+ and SOM+ Inhibitory Cells

Neuronal excitability within neural circuits is essentially regulated via cell-autonomous mechanisms (e.g., intrinsic excitability) and through the balance of excitatory (glutamatergic) and inhibitory (GABAergic) synaptic inputs. Inhibitory interneurons do not only enable the homeostatic regulation of neuronal excitability, but are likewise involved in regulating information processing and neuronal plasticity underlying the encoding and formation of specific memories (e.g., Hensch, 2005; Levelt and Hübener, 2012; Lovett-Barron et al., 2012; Liguz-Lecznar et al., 2016; Scheyltjens and Arckens, 2016). Thus, as to the homeostatic regulation of cortical circuit activity, the parallel *in vivo* imaging of excitatory and inhibitory postsynaptic sites on the same neuron revealed a matched turnover of excitatory and inhibitory synapses, whereby the turnover of inhibitory synapses occurs at a shorter timescale than that of excitatory synapses (Hayama et al., 2013; Villa et al., 2016). On the other side, inhibitory activity of GABAergic interneurons has been shown, for example, to enforce precise timing of action potentials during memory encoding (Wehr and Zador, 2003; Higley, 2006; Isaacson and Scanziani, 2011).

The two-fold function of inhibitory neurons contributing to the excitation/inhibition balance at the circuit and network level, and simultaneously supporting synaptic plasticity underlying the formation of specific memories might partly be owed to the central role these cells play in the generation of brain rhythms. In particular, fast-spiking PV+ interneurons are thought to be critically involved in the generation of major brain oscillations during sleep, including spindles, hippocampal ripples during SWS and theta activity during REM sleep (Peyrache et al., 2011; Royer et al., 2012; Amilhon et al., 2015; Averkin et al., 2016; Ognjanovski et al., 2017).

Against this backdrop we have started a series of studies aiming to characterize the excitation/inhibition balance during sleep using *in vivo* two-photon imaging of cortical circuits in mice. We simultaneously monitored Ca^2+^ activity in PV+ and SOM+ cells, respectively and neighboring pyramidal-like (Pyr) cells in cortical layers 2 and 3. Although there is a great variety of interneurons in the cortex, these cells represent the great majority of such cells, with PV+ interneurons constituting ~40% and SOM+ interneurons constituting ~30% of all cortical interneurons (Douglas and Martin, 2004; Markram et al., 2004; Rudy et al., 2011). These two classes of cells themselves also represent rather heterogeneous cell populations targeting a wide range of different cells in different layers (Tremblay et al., 2016; Miyamae et al., 2017). Thus, within the class of PV+ neurons basket cells are most frequent in the neocortex but, chandelier cells also occur in considerable numbers with projections typically targeting many more pyramidal cells than those of the basket cells which is likely associated with different functions (Klausberger and Somogyi, 2008). However, despite of this heterogeneity a feature hallmarking the majority of PV+ cells is that their projection target the perisomatic region of pyramidal cells (including the soma and axon hill) which makes them quite distinct from the SOM+ cells which more densely innervate the distal and apical portions of the dendrite (Douglas and Martin, 2004; Markram et al., 2004; Kubota et al., 2016; Yavorska and Wehr, 2016). In addition, the cells appear to be differentially regulated by neuromodulators with SOM+ but not PV+ cell activity being facilitated by acetylcholine and noradrenaline (Kawaguchi, 1997; Kawaguchi and Shindou, 1998; Muñoz and Rudy, 2014). This is of relevance inasmuch as activity of these neuromodulators distinctly differs between SWS and REM sleep, with cholinergic tone reaching minimum levels during SWS but a maximum during REM sleep. Conversely, there is considerable noradrenergic activity during SWS linked to the slow oscillation (Eschenko et al., 2012; Logothetis et al., 2012) but minimum noradrenergic activity during REM sleep.

**Figure 2:**
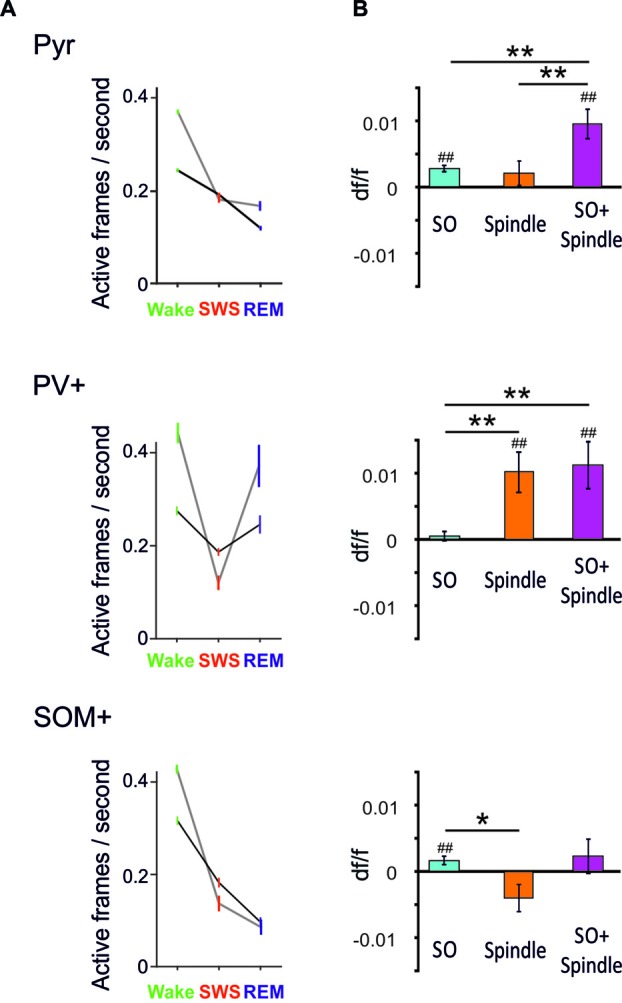
**(A)** Mean (±SEM) activity during epochs of wakefulness, SWS and REM sleep is shown for pyramidal-like cells (top), PV+ cells (middle) and SOM+ cells (bottom). Black lines—activity across all cells, gray lines—activity of the 20% cells most active during the wake phases. Note, compared with SWS, REM sleep is characterized by distinctly increased PV+ cell activity while SOM+ cell activity reaches minimum levels. This dynamics is particularly pronounced in the wake active cells (data are from Niethard et al., 2016, with permission from Elsevier). **(B)** Mean (±SEM) activity during the slow oscillation upstate (with reference to activity during a baseline interval −3 to −2 s before the event) for slow oscillations (SO), spindles (Spindle), and spindles that nested in a slow oscillation upstate (SO+ Spindle). ^##^*p* < 0.01, for comparisons against baseline activity; **p < 0.01, *p < 0.05, for pairwise comparisons. Note, spindles and spindles nesting in a slow oscillation upstates are characterized by high PV+ cell activity in the presence of low SOM+ cell activity. Spindles co-occuring with SO are additionally associated with increased pyramidal cell activity (Niethard et al., unpublished).

By comparing averaged Ca^2+^ activity during wakefulness, SWS and REM sleep epochs, in a first study (Niethard et al., 2016), we found that both sleep stages were accompanied by a distinct decrease in Pyr cell activity relative to wakefulness; however during SWS, such decrease was paralleled by reduced PV+ and SOM+ cell activity (Niethard et al., 2016; Figure 2). Remarkably, REM sleep was characterized by minimum Pyr cell activity, which was accompanied by a selective increase in the activity of PV+ cells, whereas the activity of SOM+ interneurons was further diminished. This pattern identified REM sleep as the brain state in which the cortical excitation/inhibition balance was shifted towards maximum inhibition. The pattern moreover suggested that this shift is achieved via increased perisomatic inhibition of Pyr cells via PV+ cells, while SOM+ cells were most inactive.

Our further *in vivo* two-photon imaging experiments focused on spindles and SO during SWS—network activity patterns which have been particularly linked to active systems consolidation processes in the hippocampus-dependent declarative memory system (Diekelmann and Born, 2010; Inostroza and Born, 2013; Dudai et al., 2015). In this systems consolidation process, the upstates of the slow oscillation drive, in parallel, thalamocortical spindles and hippocampal ripples with the latter accompanying replay of newly-encoded episodic information in the hippocampus. This parallel drive enables the formation of spindle-ripple events that are thought to facilitate the transmission of reactivated hippocampal memory information towards neocortical networks for long-term storage. In this perspective, spindles occurring during the excitable upstate of the slow oscillation are expected, via increasing Ca^2+^ in pyramidal cells, to be especially effective in inducing persisting plastic synaptic changes that underlie the formation of consolidated memory presentations in neocortical networks (Sejnowski and Destexhe, 2000; Ruch et al., 2012; Latouchmane et al., 2017). Imaging Ca^2+^ activity during SO and spindles revealed that both slow oscillation upstates and, less distinctly, spindles are associated with increased Pyr cell activity (Figure 2, Niethard et al., unpublished). Spindles were additionally characterized by a profound increase in PV+ interneuron activity. Consistent with the systems consolidation concept discussed above, highest levels of Pyr cell activity were observed during spindles that nested in a slow oscillation upstate. Interestingly, this maximum activity of excitatory Pyr cells was accompanied by a specific pattern of inhibitory interneuron activity comprising high activity of PV+ cells but overall low activity of SOM+ cells (Figure 2).

Taken together, these first insights into the cell-type specificity of cortical circuit activity during sleep identify a motif of high perisomatic inhibition of pyramidal cells (mediated by PV+ cells) and concomitantly low levels of dendritic inhibition (mediated by SOM+ interneurons) which is similarly observed during REM sleep and slow oscillation-spindle events in SWS. During slow oscillation-spindle events such inhibitory dynamics occur in the presence of maximum pyramidal cell activity, which might effectively support the upregulation of select synapse formation through local dendritic calcium influx and, thereby, the consolidation of memories. During REM sleep, the same inhibitory constellation of pyramidal cells accompanied by lowest activity of pyramidal cells might support global synaptic down-scaling. In fact, a shift towards predominant PV+ cell mediated perisomatic inhibition of pyramidal cells is also observed during learning: in mice, after learning a motor task, new spines grew locally on the apical dendrites of layer 2/3 pyramidal cells while axonal boutons of SOM+ interneurons were eliminated. At the same time, PV+ interneurons showed increased numbers of axonal boutons (Chen et al., 2015). Thus, the shift towards predominant PV+ cell-mediated perisomatic inhibition together with a release of distal dendrites from inhibition by SOM+ cells, might represent a general motif favoring synaptic plasticity in pyramidal cell networks across sleep and wake states.

## Conclusion

Sleep serves a dual function in regulating cortical network plasticity, i.e., to locally strengthen circuits that represent freshly encoded memories, and to globally re-normalize synaptic connectivity according to homeostatic principles. Here we covered recent studies addressing the question of how cortical circuits might establish these functions during sleep in a coherent way. While currently available data differ remarkably in respect to the parameters, the cortical area and the layers analyzed, an emerging picture becomes already apparent: structural analysis of synapses, electrophysiological studies of neural spiking activity and Ca^2+^ imaging data support the view that, on average, sleep leads to a down-scaling of synaptic connectivity and neural excitability in cortical networks. Such global down-scaling can be considered a reflection of the homeostatic regulation of network connectivity in the frame of the sleep/wake cycle. There is also consistent evidence from these studies that activity in selected subgroups of synapses and neurons is upregulated across sleep, with this upregulation possibly reflecting the enhancing effect of sleep on specific memories (Yang et al., 2014). Whereas global processes of downscaling have been linked to REM sleep (Born and Feld, 2012; Li et al., 2017), the upscaling of selected synapses has been preferentially observed during SWS (Yang et al., 2014). These fundamental differences highlight the need for future studies performing sleep stage specific analyses.

The underlying plastic processes during SWS and REM sleep, respectively, might share some basic mechanisms. Slow oscillation-spindle events represent the EEG signatures of memory related plasticity during SWS, whereas during REM sleep plasticity has been mainly linked to the pervasive EEG theta activity during this state. Ca^2+^ imaging studies revealed that both of these EEG signatures, slow oscillation-spindle events during SWS and REM sleep theta concur with a shift towards increased perisomatic inhibition of cortical pyramidal cells (via PV+ interneurons) and simultaneously diminished dendritic inhibition of the cells (via SOM+ interneurons). This constellation of pyramidal cell inhibition might favor plasticity in both directions, towards synaptic growth and potentiation during SWS as well as towards synaptic pruning and diminished neural excitability during REM sleep.

The specific brain oscillations might actively cooperate to favor plasticity. PV+ interneurons are not only involved in the generation of spindles and theta oscillations, but the perisomatic inhibition these cells impose on pyramidal cells is highly modulated by these rhythms (e.g., Wulff et al., 2009; Peyrache et al., 2011). During SWS, inputs carrying reactivated memory information, nested within the excitable troughs of the ~12-Hz spindle cycle, might reach the distal dendrites of cortical pyramidal cells; the concurrent oscillation of the cell’s membrane potential via PV-mediated perisomatic inhibition might provide temporal windows for coincidence detection and hence the induction of spike-dependent plasticity mechanisms (Peyrache et al., 2011; Averkin et al., 2016). Indeed, input firing patterns during spindles have been found to effectively induce synaptic potentiation in cortical neurons (Rosanova and Ulrich, 2005). While these spindle-related processes can also lead to an increase in principal cell firing rates—thus implicating increased excitability—homeostatic counter-regulatory processes are initialized for maintaining the excitation/inhibition balance within the involved circuits at different scales in time and space. At the circuit level, mutual inhibition between PV+ and SOM+ cells might help balance the cell’s excitability along its somatodendritic axis (Pfeffer et al., 2013; Wolff et al., 2014), and at the network level global downscaling during REM sleep following SWS might be considered to also counter local synaptic upregulation induced during SWS spindles (Li et al., 2017). One might speculate that theta oscillations during REM sleep, in a similar co-operative manner, through the rhythmic activation of PV+ cells, provides windows that support synaptic depotentiation and global downscaling (Booth and Poe, 2006; Wulff et al., 2009). This raises, of course the intriguing question, why REM theta, opposite to wake theta or SWS spindle activity, facilitates synaptic downscaling. In fact, while we here propose the interplay of brain rhythms with the reciprocal inhibitory control of pyramidal cell activity via PV+ and SOM+ cells as a general mechanism that mediates plasticity during sleep, the factors deciding about the direction of this plasticity remain to be elucidated.

## Author Contributions

NN: conception and draft of the article; figure panels. AB: revision of the article. JB: conception and revision of the article.

## Conflict of Interest Statement

The authors declare that the research was conducted in the absence of any commercial or financial relationships that could be construed as a potential conflict of interest.

## References

[B52] Amilhon B. Huh C. Y. L. Manseau F. Ducharme G. Nichol H. Adamantidis A.. (2015). Parvalbumin interneurons of hippocampus tune population activity at theta frequency. Neuron 86, 1277–1289. 10.1016/j.neuron.2015.05.027 26050044

[B1] Aton S. J. Seibt J. Dumoulin M. Jha S. K. Steinmetz N. Coleman T.. (2009). Mechanisms of sleep-dependent consolidation of cortical plasticity. Neuron 61, 454–466. 10.1016/j.neuron.2009.01.007 19217381 PMC2665998

[B2] Averkin R. G. Szemenyei V. Bordé S. Tamás G. (2016). Identified cellular correlates of neocortical ripple and high-gamma oscillations during spindles of natural sleep. Neuron 92, 916–928. 10.1016/j.neuron.2016.09.032 27746131 PMC5130902

[B3] Badura A. Sun X. R. Giovannucci A. Lynch L. A. Wang S. S.-H. (2014). Fast calcium sensor proteins for monitoring neural activity. Neurophotonics 1:25008. 10.1117/1.nph.1.2.025008 25558464 PMC4280659

[B4] Béïque J.-C. Lin D.-T. Kang M.-G. Aizawa H. Takamiya K. Huganir R. L. (2006). Synapse-specific regulation of AMPA receptor function by PSD-95. Proc. Natl. Acad. Sci. U S A 103, 19535–19540. 10.1073/pnas.0608492103 17148601 PMC1748260

[B5] Booth V. Poe G. R. (2006). Input source and strength influences overall firing phase of model hippocampal CA1 pyramidal cells during theta: relevance to REM sleep reactivation and memory consolidation. Hippocampus 16, 161–173. 10.1002/hipo.20143 16411243 PMC1401491

[B6] Born J. Feld G. B. (2012). Sleep to upscale, sleep to downscale: balancing homeostasis and plasticity. Neuron 75, 933–935. 10.1016/j.neuron.2012.09.007 22998858

[B7] Bushey D. Tononi G. Cirelli C. (2011). Sleep and synaptic homeostasis: structural evidence in Drosophila. Science 332, 1576–1581. 10.1126/science.1202839 21700878 PMC3128387

[B8] Chen S. X. Kim A. N. Peters A. J. Komiyama T. (2015). Subtype-specific plasticity of inhibitory circuits in motor cortex during motor learning. Nat. Neurosci. 18, 1109–1115. 10.1038/nn.4049 26098758 PMC4519436

[B9] Chen T.-W. Wardill T. J. Sun Y. Pulver S. R. Renninger S. L. Baohan A.. (2013). Ultrasensitive fluorescent proteins for imaging neuronal activity. Nature 499, 295–300. 10.1038/nature12354 23868258 PMC3777791

[B10] Cichon J. Gan W.-B. (2015). Branch-specific dendritic Ca^2+^ spikes cause persistent synaptic plasticity. Nature 520, 180–185. 10.1038/nature14251 25822789 PMC4476301

[B11] Civardi C. Boccagni C. Vicentini R. Bolamperti L. Tarletti R. Varrasi C.. (2001). Cortical excitability and sleep deprivation: a transcranial magnetic stimulation study. J. Neurol. Neurosurg. Psychiatry 71, 809–812. 10.1136/jnnp.71.6.809 11723210 PMC1737655

[B12] de Vivo L. Bellesi M. Marshall W. Bushong E. A. Ellisman M. H. Tononi G.. (2017). Ultrastructural evidence for synaptic scaling across the wake/sleep cycle. Science 355, 507–510. 10.1126/science.aah5982 28154076 PMC5313037

[B13] Del Cid-Pellitero E. Plavski A. Mainville L. Jones B. E. (2017). Homeostatic changes in GABA and Glutamate receptors on excitatory cortical neurons during sleep deprivation and recovery. Front. Syst. Neurosci. 11:17. 10.3389/fnsys.2017.00017 28408870 PMC5374161

[B14] Diekelmann S. Born J. (2010). The memory function of sleep. Nat. Rev. Neurosci. 11, 114–126. 10.1038/nrn2762 20046194

[B15] Diering G. H. Nirujogi R. S. Roth R. H. Worley P. F. Pandey A. Huganir R. L. (2017). Homer1a drives homeostatic scaling-down of excitatory synapses during sleep. Science 355, 511–515. 10.1126/science.aai8355 28154077 PMC5382711

[B16] Douglas R. J. Martin K. A. C. (2004). Neuronal circuits of the neocortex. Annu. Rev. Neurosci. 27, 419–451. 10.1146/annurev.neuro.27.070203.144152 15217339

[B17] Dudai Y. Karni A. Born J. (2015). The consolidation and transformation of memory. Neuron 88, 20–32. 10.1016/j.neuron.2015.09.004 26447570

[B19] Eschenko O. Magri C. Panzeri S. Sara S. J. (2012). Noradrenergic neurons of the locus coeruleus are phase locked to cortical up-down states during sleep. Cereb. Cortex 22, 426–435. 10.1093/cercor/bhr121 21670101

[B102] Frankland P. W. O’Brien C. Ohno M. Kirkwood A. Silva A. J. (2001). α-CaMKII-dependent plasticity in the cortex is required for permanent memory. Nature 411, 309–313. 10.1038/35077089 11357133

[B20] Grosmark A. D. Buzsáki G. (2016). Diversity in neural firing dynamics supports both rigid and learned hippocampal sequences. Science 351, 1440–1443. 10.1126/science.aad1935 27013730 PMC4919122

[B21] Grosmark A. D. Mizuseki K. Pastalkova E. Diba K. Buzsáki G. (2012). REM sleep reorganizes hippocampal excitability. Neuron 75, 1001–1007. 10.1016/j.neuron.2012.08.015 22998869 PMC3608095

[B22] Hashmi A. Nere A. Tononi G. (2013). Sleep-dependent synaptic down-selection (II): single-neuron level benefits for matching, selectivity, and specificity. Front. Neurol. 4:148. 10.3389/fneur.2013.00148 24151486 PMC3790262

[B23] Hayama T. Noguchi J. Watanabe S. Takahashi N. Hayashi-Takagi A. Ellis-Davies G. C. R.. (2013). GABA promotes the competitive selection of dendritic spines by controlling local Ca^2+^ signaling. Nat. Neurosci. 16, 1409–1416. 10.1038/nn.3496 23974706 PMC4135703

[B24] Hengen K. B. Torrado Pacheco A. McGregor J. N. Van Hooser S. D. Turrigiano G. G. (2016). Neuronal firing rate homeostasis is inhibited by sleep and promoted by wake. Cell 165, 180–191. 10.1016/j.cell.2016.01.046 26997481 PMC4809041

[B25] Hensch T. K. (2005). Critical period plasticity in local cortical circuits. Nat. Rev. Neurosci. 6, 877–888. 10.1038/nrn1787 16261181

[B26] Higley M. J. (2006). Balanced excitation and inhibition determine spike timing during frequency adaptation. J. Neurosci. 26, 448–457. 10.1523/JNEUROSCI.3506-05.2006 16407542 PMC6674406

[B27] Hofer S. B. Mrsic-Flogel T. D. Bonhoeffer T. Hübener M. (2009). Experience leaves a lasting structural trace in cortical circuits. Nature 457, 313–317. 10.1038/nature07487 19005470 PMC6485433

[B28] Huber R. Deboer T. Tobler I. (2000). Topography of EEG dynamics after sleep deprivation in mice. J. Neurophysiol. 84, 1888–1893. 11024081 10.1152/jn.2000.84.4.1888

[B29] Huber R. Mäki H. Rosanova M. Casarotto S. Canali P. Casali A. G.. (2013). Human cortical excitability increases with time awake. Cereb. Cortex 23, 332–338. 10.1093/cercor/bhs014 22314045 PMC3539451

[B30] Inostroza M. Born J. (2013). Sleep for preserving and transforming episodic memory. Annu. Rev. Neurosci. 36, 79–102. 10.1146/annurev-neuro-062012-170429 23642099

[B31] Isaacson J. S. Scanziani M. (2011). How inhibition shapes cortical activity. Neuron 72, 231–243. 10.1016/j.neuron.2011.09.027 22017986 PMC3236361

[B32] Kawaguchi Y. (1997). Selective cholinergic modulation of cortical GABAergic cell subtypes. J. Neurophysiol. 78, 1743–1747. 9310461 10.1152/jn.1997.78.3.1743

[B33] Kawaguchi Y. Shindou T. (1998). Noradrenergic excitation and inhibition of GABAergic cell types in rat frontal cortex. J. Neurosci. 18, 6963–6976. 9712665 10.1523/JNEUROSCI.18-17-06963.1998 PMC6792977

[B34] Kirkpatrick J. Pascanu R. Rabinowitz N. Veness J. Desjardins G. Rusu A. A.. (2017). Overcoming catastrophic forgetting in neural networks. Proc. Natl. Acad. Sci. U S A 114, 3521–3526. 10.1073/pnas.1611835114 28292907 PMC5380101

[B101] Klausberger T. Somogyi P. (2008). Neuronal diversity and temporal dynamics: the unity of hippocampal circuit operations. Science 321, 53–57. 10.1126/science.1149381 18599766 PMC4487503

[B35] Korf E. M. Mölle M. Born J. Ngo H. V. (2017). Blindfolding during wakefulness causes decrease in sleep slow wave activity. Physiol. Rep. 5:e13239. 10.14814/phy2.13239 28408638 PMC5392525

[B36] Kreuzer P. Langguth B. Popp R. Raster R. Busch V. Frank E.. (2011). Reduced intra-cortical inhibition after sleep deprivation: a transcranial magnetic stimulation study. Neurosci. Lett. 493, 63–66. 10.1016/j.neulet.2011.02.044 21352891

[B37] Kubota Y. Karube F. Nomura M. Kawaguchi Y. (2016). The diversity of cortical inhibitory synapses. Front. Neural Circuits 10:27. 10.3389/fncir.2016.00027 27199670 PMC4842771

[B38] Lanté F. Toledo-Salas J.-C. Ondrejcak T. Rowan M. J. Ulrich D. (2011). Removal of synaptic Ca^2+^-permeable AMPA receptors during sleep. J. Neurosci. 31, 3953–3961. 10.1523/JNEUROSCI.3210-10.2011 21411638 PMC6623525

[B39] Latouchmane C.-F. V. Ngo H.-V. V. Born J. Shin H.-S. (2017). Thalamic spindles promote memory formation during sleep through triple phase-locking of cortical, thalamic, and hippocampal rhythms. Neuron 95, 424.e6–435.e6. 10.1016/j.neuron.2017.06.025 28689981

[B40] Lee H.-K. Kirkwood A. (2011). AMPA receptor regulation during synaptic plasticity in hippocampus and neocortex. Semin. Cell Dev. Biol. 22, 514–520. 10.1016/j.semcdb.2011.06.007 21856433 PMC3190053

[B41] Levelt C. N. Hübener M. (2012). Critical-period plasticity in the visual cortex. Annu. Rev. Neurosci. 35, 309–330. 10.1146/annurev-neuro-061010-113813 22462544

[B42] Li W. Ma L. Yang G. Gan W.-B. (2017). REM sleep selectively prunes and maintains new synapses in development and learning. Nat. Neurosci. 20, 427–437. 10.1038/nn.4479 28092659 PMC5535798

[B43] Liguz-Lecznar M. Urban-Ciecko J. Kossut M. (2016). Somatostatin and somatostatin-containing neurons in shaping neuronal activity and plasticity. Front. Neural Circuits 10:48. 10.3389/fncir.2016.00048 27445703 PMC4927943

[B44] Logothetis N. K. Eschenko O. Murayama Y. Augath M. Steudel T. Evrard H. C.. (2012). Hippocampal-cortical interaction during periods of subcortical silence. Nature 491, 547–553. 10.1038/nature11618 23172213

[B45] Lovett-Barron M. Turi G. F. Kaifosh P. Lee P. H. Bolze F. Sun X. H.. (2012). Regulation of neuronal input transformations by tunable dendritic inhibition. Nat. Neurosci. 15, 423–430, S1–S3. 10.1038/nn.3024 22246433

[B46] Maret S. Faraguna U. Nelson A. B. Cirelli C. Tononi G. (2011). Sleep and waking modulate spine turnover in the adolescent mouse cortex. Nat. Neurosci. 14, 1418–1420. 10.1038/nn.2934 21983682 PMC3203346

[B47] Markram H. Toledo-Rodriguez M. Wang Y. Gupta A. Silberberg G. Wu C. (2004). Interneurons of the neocortical inhibitory system. Nat. Rev. Neurosci. 5, 793–807. 10.1038/nrn1519 15378039

[B48] Matsuzaki M. Ellis-Davies G. C. Nemoto T. Miyashita Y. Iino M. Kasai H. (2001). Dendritic spine geometry is critical for AMPA receptor expression in hippocampal CA1 pyramidal neurons. Nat. Neurosci. 4, 1086–1092. 10.1038/nn736 11687814 PMC4229049

[B49] Miyamae T. Chen K. Lewis D. A. Gonzalez-Burgos G. (2017). Distinct physiological maturation of parvalbumin-positive neuron subtypes in mouse prefrontal cortex. J. Neurosci. 37, 4883–4902. 10.1523/JNEUROSCI.3325-16.2017 28408413 PMC5426180

[B50] Miyawaki H. Diba K. (2016). Regulation of hippocampal firing by network oscillations during sleep. Curr. Biol. 26, 893–902. 10.1016/j.cub.2016.02.024 26972321 PMC4821660

[B51] Muñoz W. Rudy B. (2014). Spatiotemporal specificity in cholinergic control of neocortical function. Curr. Opin. Neurobiol. 26, 149–160. 10.1016/j.conb.2014.02.015 24637201 PMC4100208

[B53] Nelson A. B. Faraguna U. Zoltan J. T. Tononi G. Cirelli C. (2013). Sleep patterns and homeostatic mechanisms in adolescent mice. Brain Sci. 3, 318–343. 10.3390/brainsci3010318 23772316 PMC3682503

[B54] Nere A. Hashmi A. Cirelli C. Tononi G. (2013). Sleep-dependent synaptic down-selection (I): modeling the benefits of sleep on memory consolidation and integration. Front. Neurol. 4:143. 10.3389/fneur.2013.00143 24137153 PMC3786405

[B55] Niethard N. Hasegawa M. Itokazu T. Oyanedel C. N. Born J. Sato T. R. (2016). Sleep-stage-specific regulation of cortical excitation and inhibition. Curr. Biol. 26, 2739–2749. 10.1016/j.cub.2016.08.035 27693142

[B56] Ognjanovski N. Schaeffer S. Wu J. Mofakham S. Maruyama D. Zochowski M.. (2017). Parvalbumin-expressing interneurons coordinate hippocampal network dynamics required for memory consolidation. Nat. Commun 6:16120. 10.1038/ncomms16120 28681846 PMC5509892

[B57] Olcese U. Esser S. K. Tononi G. (2010). Sleep and synaptic renormalization: a computational study. J. Neurophysiol. 104, 3476–3493. 10.1152/jn.00593.2010 20926617 PMC3007640

[B58] Peyrache A. Battaglia F. P. Destexhe A. (2011). Inhibition recruitment in prefrontal cortex during sleep spindles and gating of hippocampal inputs. Proc. Natl. Acad. Sci. U S A 108, 17207–17212. 10.1073/pnas.1103612108 21949372 PMC3193185

[B59] Pfeffer C. K. Xue M. He M. Huang Z. J. Scanziani M. (2013). Inhibition of inhibition in visual cortex: the logic of connections between molecularly distinct interneurons. Nat. Neurosci. 16, 1068–1076. 10.1038/nn.3446 23817549 PMC3729586

[B60] Piantoni G. Poil S. S. Linkenkaer-Hansen K. Verweij I. M. Ramautar J. R. Van Someren E. J.. (2013). Individual differences in white matter diffusion affect sleep oscillations. J. Neurosci. 33, 227–233. 10.1523/JNEUROSCI.2030-12.2013 23283336 PMC6618630

[B61] Placidi F. Zannino S. Albanese M. Romigi A. Izzi F. Marciani M. G.. (2013). Increased cortical excitability after selective REM sleep deprivation in healthy humans: a transcranial magnetic stimulation study. Sleep Med. 14, 288–292. 10.1016/j.sleep.2012.11.020 23343775

[B62] Rasch B. Born J. (2013). About sleep’s role in memory. Physiol. Rev. 93, 681–766. 10.1152/physrev.00032.2012 23589831 PMC3768102

[B63] Riedner B. A. Vyazovskiy V. V. Huber R. Massimini M. Esser S. Murphy M.. (2007). Sleep homeostasis and cortical synchronization: III. A high-density EEG study of sleep slow waves in humans. Sleep 30, 1643–1657. 10.1093/sleep/30.12.1643 18246974 PMC2276133

[B64] Rosanova M. Ulrich D. (2005). Pattern-specific associative long-term potentiation induced by a sleep spindle-related spike train. J. Neurosci. 25, 9398–9405. 10.1523/JNEUROSCI.2149-05.2005 16221848 PMC6725710

[B65] Royer S. E. B. Zemelman B. V. Losonczy A. Kim J. Chance F. Magee J. C.. (2012). Control of timing, rate and bursts of hippocampal place cells by dendritic and somatic inhibition. Nat. Neurosci. 15, 769–775. 10.1038/nn.3077 22446878 PMC4919905

[B66] Ruch S. Markes O. Duss S. B. Oppliger D. Reber T. P. Koenig T.. (2012). Sleep stage II contributes to the consolidation of declarative memories. Neuropsychologia 50, 2389–2396. 10.1016/j.neuropsychologia.2012.06.008 22750121

[B67] Rudy B. Fishell G. Lee S. Hjerling-Leffler J. (2011). Three groups of interneurons account for nearly 100% of neocortical GABAergic neurons. Dev. Neurobiol. 71, 45–61. 10.1002/dneu.20853 21154909 PMC3556905

[B68] Scheyltjens I. Arckens L. (2016). The current status of somatostatin-interneurons in inhibitory control of brain function and plasticity. Neural Plast. 2016:8723623. 10.1155/2016/8723623 27403348 PMC4923604

[B69] Sejnowski T. J. Destexhe A. (2000). Why do we sleep? Brain Res. 886, 208–223. 10.1016/S0006-8993(00)03007-9 11119697

[B70] Shepherd J. D. (2012). Memory, plasticity and sleep—a role for calcium permeable AMPA receptors? Front. Mol. Neurosci 5:49. 10.3389/fnmol.2012.00049 22514518 PMC3324118

[B71] Tononi G. Cirelli C. (2014). Sleep and the price of plasticity: from synaptic and cellular homeostasis to memory consolidation and integration. Neuron 81, 12–34. 10.1016/j.neuron.2013.12.025 24411729 PMC3921176

[B72] Tremblay R. Lee S. Rudy B. (2016). GABAergic interneurons in the neocortex: from cellular properties to circuits. Neuron 91, 260–292. 10.1016/j.neuron.2016.06.033 27477017 PMC4980915

[B73] Turrigiano G. (2012). Homeostatic synaptic plasticity: local and global mechanisms for stabilizing neuronal function. Cold Spring Harb. Perspect. Biol. 4:a005736. 10.1101/cshperspect.a005736 22086977 PMC3249629

[B74] Turrigiano G. G. (2017). The dialectic of Hebb and homeostasis. Philos. Trans. R. Soc. Lond. B Biol. Sci. 372:20160258. 10.1098/rstb.2016.0258 28093556 PMC5247594

[B75] Turrigiano G. G. Leslie K. R. Desai N. S. Rutherford L. C. Nelson S. B. (1998). Activity-dependent scaling of quantal amplitude in neocortical neurons. Nature 391, 892–896. 10.1038/36103 9495341

[B76] Villa K. L. Berry K. P. Subramanian J. Cha J. W. Oh W. C. Kwon H. B.. (2016). Inhibitory synapses are repeatedly assembled and removed at persistent sites *in vivo*. Neuron 89, 756–769. 10.1016/j.neuron.2016.01.010 26853302 PMC4760889

[B77] Vyazovskiy V. V. Cirelli C. Pfister-Genskow M. Faraguna U. Tononi G. (2008). Molecular and electrophysiological evidence for net synaptic potentiation in wake and depression in sleep. Nat. Neurosci. 11, 200–208. 10.1038/nn2035 18204445

[B78] Vyazovskiy V. V. Cirelli C. Tononi G. (2011). Electrophysiological correlates of sleep homeostasis in freely behaving rats. Prog. Brain Res. 193, 17–38. 10.1016/b978-0-444-53839-0.00002-8 21854953 PMC3160719

[B79] Vyazovskiy V. V. Olcese U. Lazimy Y. M. Faraguna U. Esser S. K. Williams J. C.. (2009). Cortical firing and sleep homeostasis. Neuron 63, 865–878. 10.1016/j.neuron.2009.08.024 19778514 PMC2819325

[B80] Watson B. O. Levenstein D. Greene J. P. Gelinas J. N. Buzsáki G. (2016). Network homeostasis and state dynamics of neocortical sleep. Neuron 90, 839–852. 10.1016/j.neuron.2016.03.036 27133462 PMC4873379

[B81] Wehr M. S. Zador A. M. (2003). Balanced inhibition underlies tuning and sharpens spike timing in auditory cortex. Nature 426, 442–446. 10.1038/nature02116 14647382

[B82] Wolff S. B. E. Gründemann J. Tovote P. Krabbe S. Jacobson G. A. Müller C.. (2014). Amygdala interneuron subtypes control fear learning through disinhibition. Nature 509, 453–458. 10.1038/nature13258 24814341

[B83] Wulff P. Ponomarenko A. A. Bartos M. Korotkova T. M. Fuchs E. C. Bähner F.. (2009). Hippocampal theta rhythm and its coupling with gamma oscillations require fast inhibition onto parvalbumin-positive interneurons. Proc. Natl. Acad. Sci. U S A 106, 3561–3566. 10.1073/pnas.0813176106 19204281 PMC2637907

[B84] Xu T. Yu X. Perlik A. J. Tobin W. F. Zweig J. A. Tennant K.. (2009). Rapid formation and selective stabilization of synapses for enduring motor memories. Nature 462, 915–919. 10.1038/nature08389 19946267 PMC2844762

[B85] Yang G. Gan W. B. (2012). Sleep contributes to dendritic spine formation and elimination in the developing mouse somatosensory cortex. Dev. Neurobiol. 72, 1391–1398. 10.1002/dneu.20996 22058046 PMC3404222

[B86] Yang G. Lai C. S. W. Cichon J. Ma L. Li W. Gan W.-B. (2014). Sleep promotes branch-specific formation of dendritic spines after learning. Science 344, 1173–1178. 10.1126/science.1249098 24904169 PMC4447313

[B87] Yang G. Pan F. Gan W.-B. (2009). Stably maintained dendritic spines are associated with lifelong memories. Nature 462, 920–924. 10.1038/nature08577 19946265 PMC4724802

[B88] Yavorska I. Wehr M. (2016). Somatostatin-expressing inhibitory interneurons in cortical circuits. Front. Neural Circuits 10:76. 10.3389/fncir.2016.00076 27746722 PMC5040712

[B89] Zito K. Scheuss V. Knott G. Hill T. Svoboda K. (2009). Rapid functional maturation of nascent dendritic spines. Neuron 61, 247–258. 10.1016/j.neuron.2008.10.054 19186167 PMC2800307

